# Does non-invasive brain stimulation reduce essential tremor? A systematic review and meta-analysis

**DOI:** 10.1371/journal.pone.0185462

**Published:** 2017-09-28

**Authors:** Nyeonju Kang, James H. Cauraugh

**Affiliations:** 1 Laboratory for Rehabilitation Neuroscience, Department of Applied Physiology and Kinesiology, University of Florida, Gainesville, Florida, United States of America; 2 Motor Behavior Laboratory, Department of Applied Physiology and Kinesiology, University of Florida, Gainesville, Florida, United States of America; 3 Division of Sport Science, Incheon National University, Incheon, South Korea; Universitair Medisch Centrum Groningen, NETHERLANDS

## Abstract

Essential tremor (ET) is the most common age-related disease leading to abnormal tremulous behaviors in the upper and lower extremities. Non-invasive brain stimulation (NIBS) may be an effective ET therapy by modulating the oscillating network of the brain. The current systematic review and meta-analysis examined the effects of NIBS interventions on tremor symptoms in ET patients. Our comprehensive search identified eight studies that used 1 Hz of rTMS, cTBS, or ctDCS protocols. Twenty total comparisons from the eight qualified studies were statistically synthesized, and the meta-analytic findings revealed that NIBS techniques reduced tremulous behaviors in individuals with ET. Moreover, the four moderator variable analyses demonstrated that the positive therapeutic effects of NIBS appeared across the following subgroups: (a) tremor assessment (clinical test vs. quantitative tremor assessment), (b) stimulation site (cerebellum vs. motor cortex), (c) session number (single session vs. multiple sessions), and (d) sustained positive treatment effect (posttest vs. retention test). This comprehensive systematic review and meta-analysis provided evidence that support positive treatment effects of NIBS techniques on ET motor therapy.

## Introduction

Essential tremor (ET) is one of the most common neurological diseases in older adults leading to abnormal tremulous behaviors in the upper and lower extremities [1]. The incidence of ET is approximately 4% of the individuals over 40 years of age and older, and continually increases with age [2, 3]. Specifically, action tremor in individuals with ET is predominantly observed in the arms [4], and the severity of tremor is occasionally progressive over time [5–7]. Treatment protocols for ET have focused on medications (i.e., primidone and propranolol) and surgery (e.g., deep brain stimulation, focused ultrasound, and thalamotomy) [2, 8]. Although various approaches report a reduction of tremors, the pharmacological and surgical interventions have several limitations including cost and potential side effects [9, 10]. Thus, the search for more effective treatment options for motor recovery of ET patients continues.

During the past decade, non-invasive brain stimulation (NIBS) techniques as alternatives to the conventional ET treatments has been investigated by ET researchers [11]. Specifically, NIBS protocols used for individuals with ET involve repetitive transcranial magnetic stimulation (rTMS), theta burst stimulation (TBS), and transcranial direct current stimulation (tDCS) [11]. A common assumption for these NIBS techniques is that delivering low electrical current to the scalp may induce either cortical excitation or inhibition in the targeted area of the brain, and this altered cortical activity presumably affects motor functions [12, 13]. Further, rTMS delivers small electrical currents in the regions of the brain using a stimulation coil producing a magnetic field near the patient’s scalp. A high frequency current (> 1 Hz) of rTMS causes cortical excitation, whereas a low frequency current (≤ 1 Hz) of rTMS induces cortical inhibition in the targeted brain area [14]. TBS, a new paradigm of TMS, delivers short bursts within the theta range (i.e., 5 Hz) in which each burst consists of three magnetic pulses delivered at a high stimulation frequency (i.e., 50 Hz). An intermittent TBS (iTBS) increases cortical activity, whereas a continuous TBS (cTBS) decreases cortical activity [15]. tDCS transfers an electric current of low intensity (i.e., 1–2 mA) to the scalp via anodal and cathodal electrodes. Anodal tDCS (atDCS) facilitates cortical activity, whereas cathodal tDCS (ctDCS) suppresses cortical activity [12].

For reducing tremor in ET patients, rTMS, TBS, and tDCS protocols commonly used an inhibition stimulation to suppress cortical activity in specific regions such as the posterior cerebellum, primary motor cortex (M1), premotor cortex, and supplementary motor area (SMA) [11]. These stimulation protocols are theoretically based on the oscillating network hypothesis that hyperactive multiple regions in the brain (i.e., tremor network) dynamically act as oscillators leading to tremor [1, 16]. The cerebello-thalamo-cortical (CTC) circuit may be one of the potential tremor network properties because of the convincing evidence that lesions at several different locations along the CTC circuit can eliminate tremulous behaviors in ET [17]. Thus, researchers hypothesize that tuning down hyperactive CTC circuit by applying NIBS on either motor cortex or cerebellum can reduce tremors in ET patients [18]. Indeed, recent narrative reviews reported evidence supporting the potential contribution of NIBS interventions to ET motor functions [11, 18–20]. However, the authors suspected that these findings are still controversial because of small sample sizes for each individual study and increased methodological heterogeneity such as tremor assessment (e.g., clinical test and quantitative tremor assessment), brain site for stimulation (e.g., cerebellum and motor cortex), the number of stimulation sessions (e.g., single session and multiple sessions), and the sustained therapeutic effect (e.g., posttest and retention test). To date, no meta-analysis study has systematically addressed the above possible issues of NIBS interventions suggested by prior studies with a meaningful overall therapeutic effect on ET patients.

Thus, the current systematic review and comprehensive meta-analysis investigated the effects of NIBS treatments including rTMS, TBS, and tDCS on reducing tremor in ET patients. We addressed four leading questions: (a) Do NIBS interventions improve tremulous behaviors as indicated by clinical test or quantitative tremor assessment? (b) Does stimulation on the cerebellum or motor cortex show improvements in tremor? (c) Does the number of stimulation sessions (i.e., single vs. multiple) influence treatment effects in ET? and (d) Do NIBS interventions exhibit a reduction in tremor at posttest (≤ 1 day) or retention test (> 1 day)?

## Materials and methods

### Literature search and study selection

The current systematic review and comprehensive meta-analysis followed the suggestions of The PRISMA statement [21]. Our computerized literature searches focused on NIBS studies that examined the effects of rTMS, TBS, or tDCS on tremor in patients with ET. The period of literature searches was January 2017 –June 2017, and we did not confine our search by the type of publications (i.e., refereed articles, letters to editor, conference proceedings, and negative results). Three databases were used for the systematic literature search: (a) PubMed, (b) ISI’s Web of Knowledge, and (c) Cochrane Database of Systematic Reviews. Keywords input for searching included: (1) rTMS: (essential tremor) AND (rTMS or repetitive transcranial magnetic stimulation), (2) TBS: (essential tremor) AND (TBS or theta burst stimulation), and (3) tDCS: (essential tremor) AND (tDCS or transcranial direct current stimulation).

**Fig 1:**
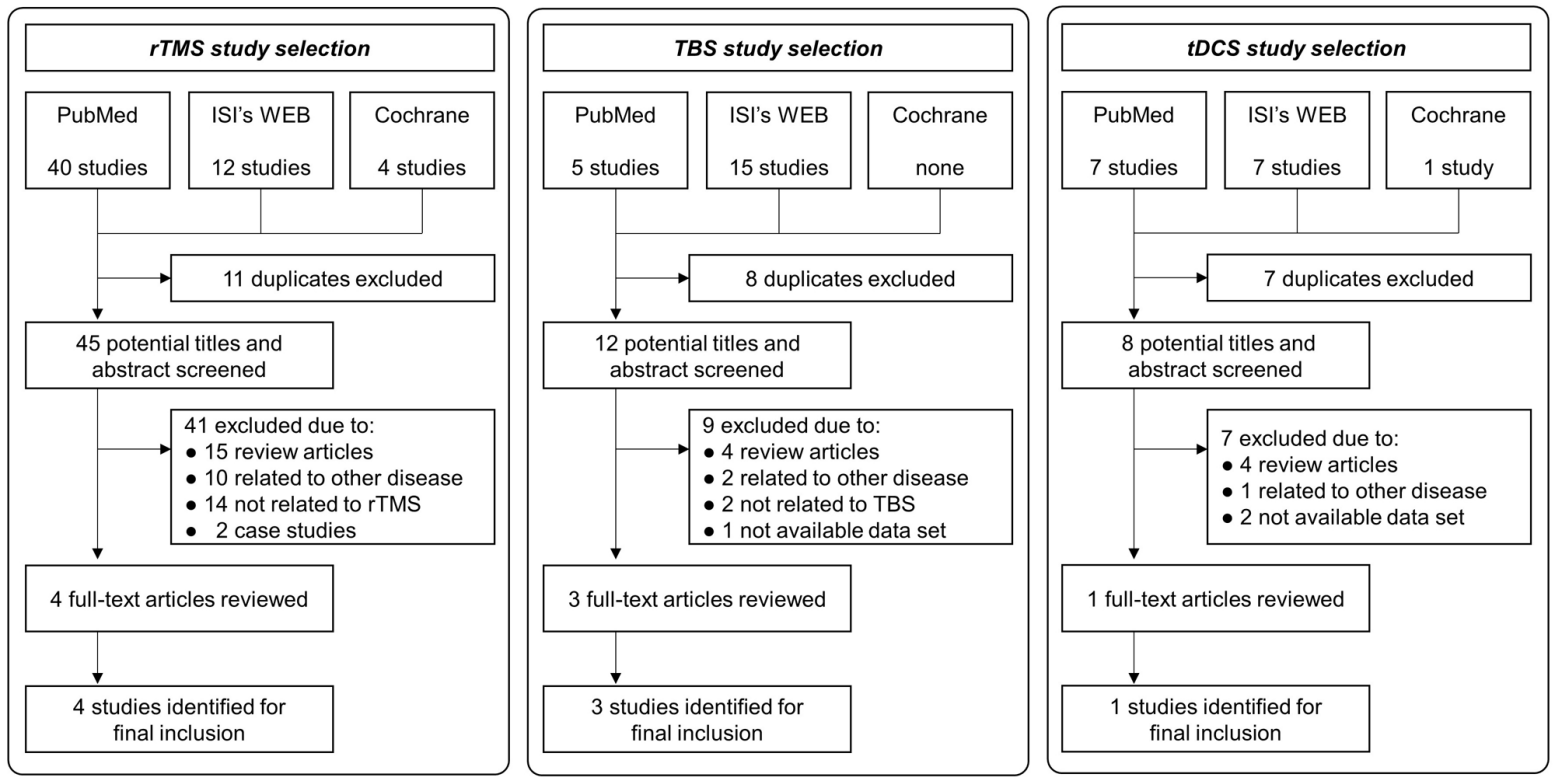
Flow chart for study selection.

Fig 1 shows the selection algorithm and numbers of included and excluded studies. To minimize bias, the authors independently reviewed titles, abstracts, and text based on our inclusion and exclusion criteria. Included studies met the following criteria: (1) quantitative evaluation of rTMS, TBS, or tDCS effects on tremor in ET patients, (2) tremor assessment using either clinical tests or quantitative tremor assessment, and (3) either a between-group comparison (active stimulation vs. sham) or a within-group comparison (pretest vs. posttest).

We excluded review papers and studies that failed to report values for calculating individual effect sizes. Based on our criteria, 91 potential publications were initially found, and the first screening excluded 26 duplicated articles. Next, substantial reviews on the 65 remaining articles identified 57 additional publications for exclusion (e.g., review articles, other disease, not related to NIBS, and case studies). The final eight high quality publications qualified for the meta-analysis [22–29].

The eight qualified studies included four rTMS (i.e., rTMS ≤ 1 Hz) studies, three TBS (i.e., continuous TBS: cTBS) studies, and one tDCS (i.e., cathodal tDCS: ctDCS) study. We analyzed multiple comparisons from each study if the comparisons were different based on tremor assessment (clinical test vs. quantitative tremor assessment), stimulation site (cerebellum vs. motor cortex), stimulation session (single session vs. multiple sessions), and sustained treatment effect (posttest: ≤ 1 day vs. retention test: > 1 day). For these qualified studies, a total of 20 comparisons were submitted to our meta-analysis: (a) 11 clinical test comparisons vs. nine quantitative tremor assessment comparisons, (b) 12 cerebellum (12 posterior cerebellum) comparisons vs. eight motor cortex (three M1, four pre-SMA, and one premotor cortex) comparisons, (c) seven single session vs. 13 multiple sessions (2–15 sessions), and (d) 13 posttest comparisons vs. seven retention test comparisons. Specifically, the 11 clinical test comparisons included: (a) six cerebellum comparisons vs. five motor cortex comparisons, (b) two single session vs. nine multiple sessions, and (c) six posttest comparisons vs. five retention test comparisons. The nine quantitative tremor assessment comparisons had: (a) six cerebellum comparisons vs. three motor cortex comparisons, (b) five single session vs. four multiple sessions, and (c) seven posttest comparisons vs. two retention test comparisons.

### Tremor outcome measurements

The primary outcome measures for estimating tremor severity of the upper limb for ET patients included: (a) 11 clinical assessment comparisons (nine FTM-TRS: the Fahn-Tolosa-Marin Tremor Rating Scale, one TETRAS: The Essential Tremor Rating Assessment Scale, and one TCRS: Tremor Clinical Rating Scale), and (b) nine quantitative tremor assessment comparisons (eight accelerometer and one motion analysis). Further, for the quantitative tremor assessment, we selected the tremor amplitude of movements quantified by accelerometer and motion analysis, commonly reported by all nine comparisons.

### Data synthesis and analysis

**Fig 2:**
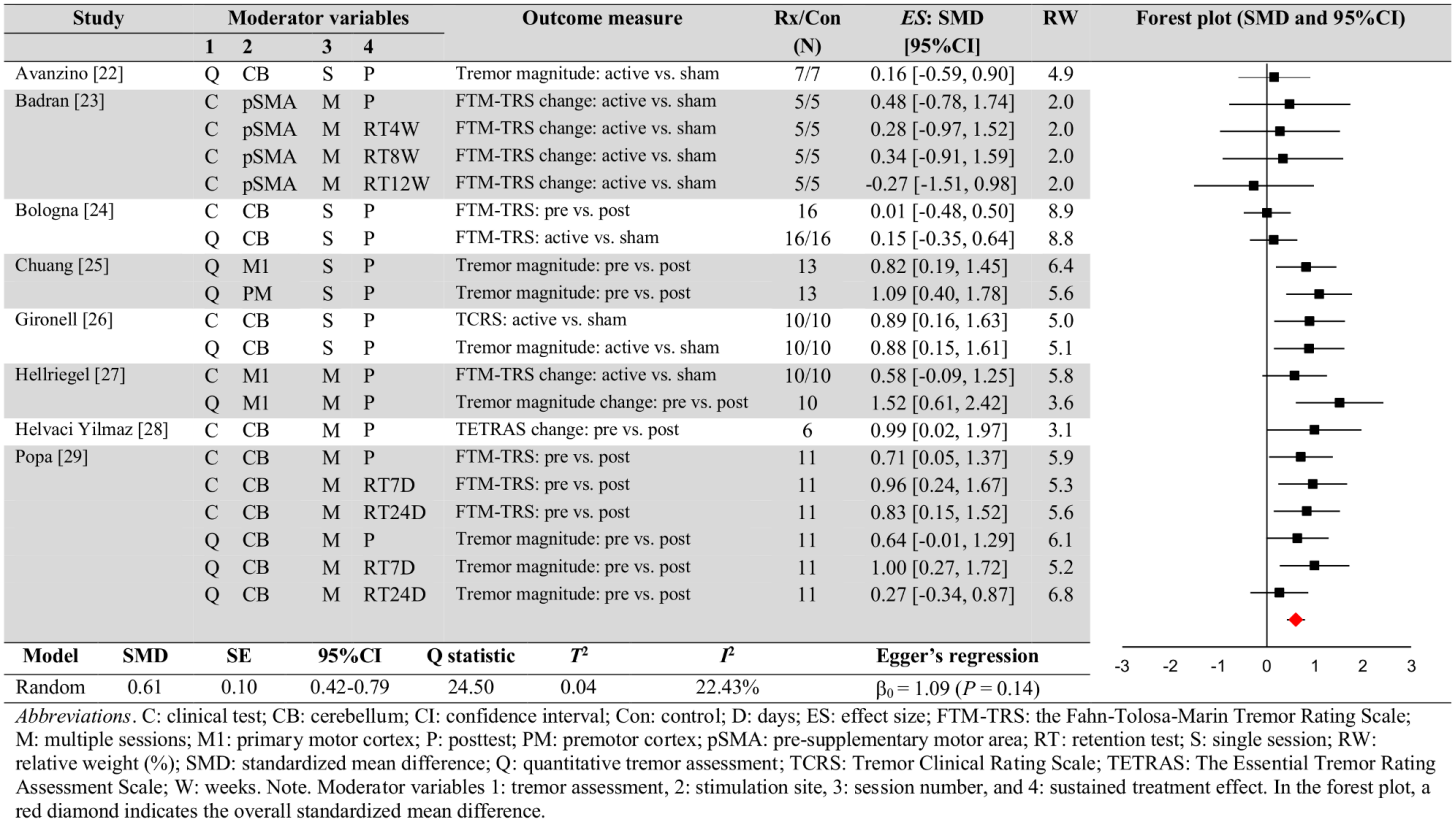
Meta-analytic findings.

Using the Comprehensive Meta-Analysis software (ver. 3.0, Englewood, NJ, USA), we calculated and determined meta-analytic findings. Individual effect size for each comparison was quantified using either (a) difference in tremor severity between the active stimulation and sham stimulation at the posttests or (b) changes in tremor severity for the active stimulation between pretest and posttest (or retention test) [30]. Importantly, we confirmed (a) no significant difference in tremor severity between the active stimulation and sham stimulation at the pretests and (b) no significant changes in tremor severity for the sham stimulation between pretests and posttests (or retention tests). Table 1 shows demographic and clinical information for the ET patients, and Tables 2 and 3 summarize the tremor assessments and specific stimulation protocols. For estimating methodological quality of each study, we reported PEDro scores in Table 4 [31]. Statistical summary data that include individual weighted effect sizes (standardized mean difference), confidence intervals, standardized effect size, *Q* statistic, *T*^2^, *I*^2^, and Egger’s regression intercept are shown in Fig 2. To calculate effect sizes from each comparison, we used mean values, sample sizes, *P*-values, and *t*-values reported in either the text or figures of included published articles. Consistent with conventional recommendations by Borenstein and colleagues [30, 32], we selected the random effects model for this meta-analysis. The authors suggested that the random effects model is appropriate when included individuals studies collected from the published literature because this model assumes that effect sizes differ as a function of some causes (e.g., participants or treatment protocols) and no common effect size appears across studies.

**Table 1:** Demographic and clinical information in individuals with essential tremor.

Study	Total N	Age (year)	Gender	TSO (year)	Tremor type	Tremor severity at baseline	Medication control
Avanzino [22]	15	56.8±10.8	10F, 10M	18.2±17.7	7H, 8S	CRSC _Part 1_: 13.3±5.1	stop before 72 hours
Badran [23]	10	71.6±12.2	6F, 4M	NR	NR	FTM-TRS _total_: 35.0±6.7	NR
Bologna [24]	16	60.8±8.3	6F, 10M	17.9±11.7	10H, 6S	FTM-TRS _R-hand (~24)_: 10.8±4.5	stop before 4 weeks
Chuang [25]	13	51.9±18.4	5F, 8M	NR	NR	NR	NR
Gironell [26]	10	67.9: 55–57	3F, 7M	12.2: 5–25	3H, 7S	TCRS _Part 1 and 2_: 24.5±12.5	stop before 72 hours
Hellriegel [27]	10	65.2±10.4	1F, 9M	37.2±22.6	7H, 3S	FTM-TRS _Item A and B (~24)_: 6.3±2.4	stop before 4 weeks
Helvaci Yilmaz [28]	6	37.2±1.9	3F, 3M	11.8±6.0	3H, 3S	TETRAS _Total_: 9.8±3.4	stop before 1 week
Popa [29]	11	51.5±11.8	3F, 8M	23.9±14.5	5H, 6S	FTM-TRS _total_: 46.0±13.0	NR

*Abbreviations*. CRSC: Clinical Rating Scale for Tremor; F: female; FTM-TRS: the Fahn-Tolosa-Marin Tremor Rating Scale; H: hereditary; M: male; NR: not reported; S: sporadic; TCRS: Tremor Clinical Rating Scale; TETRAS: The Essential Tremor Rating Assessment Scale

**Table 2:** Tremor testing.

Study	Tremor test		Test timing
	Clinical	Quantitative tremor assessment	
Avanzino [22]	NR	Accelerometer on the right index finger Postural tremor for 60 s Tremor magnitude: square root of total power within the spectral peak of wrist tremor	Posttest
Badran [23]	FTM-TRS total	NR	Posttest Retention: 4, 8, and 12 week
Bologna [24]	FTM-TRS Right hand Scale: 0–24	Motion system analysis on the right index finger Postural tremor Tremor magnitude: units of finger displacement	Posttest
Chuang [25]	NR	Accelerometer on the index finger Action tremor during forearm extension Tremor magnitude: units of finger displacement	Posttest
Gironell [26]	TCRS Part 1 and 2	Accelerometer on the most affected index finger Postural tremor for 60 s Tremor magnitude: absolute power of the dominant frequency peak in mV squared	Posttest
Hellriegel [27]	FTM-TRS Item A and B	Accelerometer on the right index finger Postural tremor for 30 s Tremor magnitude: total power	Posttest
Helvaci Yilmaz [28]	TETRAS	NR	Posttest
Popa [29]	FTM-TRS total	Accelerometer on the most affected index finger Action tremor during wrist and fingers extension for 30 s Tremor magnitude: amplitude of the tremor peak under a Gaussian fit of the power spectrum	Posttest Retention: 7 and 24 days

*Abbreviations*. FTM-TRS: the Fahn-Tolosa-Marin Tremor Rating Scale; NR: not reported; TCRS: Tremor Clinical Rating Scale; TETRAS: The Essential Tremor Rating Assessment Scale

**Table 3:** NIBS intervention protocols.

Study	Stimulation protocol			
	Type	Session	Brain site	Parameter setup
Avanzino [22]	rTMS	1	Right posterior cerebellum 3 cm lateral and 1 cm beneath the inion	1 Hz-rTMS; 90% of RMT; 10 mins; 600 pulses Sham
Badran [23]	rTMS	15	Pre-SMA 50% of distance between EEG positions Fz and FCz	1 Hz-rTMS; 110% of RMT; 20 mins; 1200 pulses Sham
Bologna [24]	cTBS	1	Right cerebellum 3 cm lateral and 1 cm beneath the inion	cTBS; 80% of AMT; 40 s-train; repeated at every 200 ms Sham over neck muscle
Chuang [25]	cTBS	1	Left M1 Premotor (dorsolateral) 2.5 cm anterior to the motor hot-spot	cTBS; 80% of AMT; 40 s-train; repeated at every 200 ms Sham (60% of AMT) on M1
Gironell [26]	rTMS	1	Posterior cerebellum on the midline and 2 cm below inion	1 Hz-rTMS; 100% of MOI; 20 mins; 300 pulses Sham
Hellriegel [27]	cTBS	2	Left M1 hand area	cTBS; 80% of AMT; two 20 s-train with 1 min a short break; repeated at every 200 ms Sham (60% of AMT) on M1
Helvaci Yilmaz [28]	tDCS	10	Prefrontal area: atDCS Posterior cerebellum (inion): ctDCS	atDCS and ctDCS; 2 mA; 20 mins; 35 m^2^ No sham
Popa [29]	rTMS	5	Bilateral posterior cerebellum lobule VIII of each hemisphere	1 Hz-rTMS; 90% of RMT; 15 mins; 900 pulses No sham

*Abbreviations*. AMT: active motor threshold; atDCS: anodal transcranial direct current stimulation; cTBS: continuous theta burst stimulation; ctDCS: cathodal transcranial direct current stimulation; M1; primary motor area; MOI: maximum output intensity; SMA: supplementary motor area; RMT: resting motor threshold; rTMS: repetitive transcranial magnetic stimulation; tDCS: transcranial direct current stimulation.

**Table 4:** Quality assessments using PEDro score.

Items	Avanzino [22]	Badran [23]	Bologna [24]	Chuang [25]	Gironell [26]	Hellriegel [27]	Helvaci Yilmaz [28]	Popa [29]
1. Eligibility criteria were specified	1	1	1	1	1	1	1	1
2. Subjects were randomly allocated to groups (in a crossover study, subjects were randomly allocated an order in which treatments were received)	1	1	1	1	1	0	0	0
3. Allocation was concealed	0	0	0	1	1	0	0	0
4. The groups were similar at baseline regarding the most important prognostic indicators	1	1	1	1	1	1	0	0
5. There was blinding of all subjects	0	1	1	0	1	0	0	1
6. There was blinding of all therapists who administered the therapy	0	1	1	0	1	1	0	1
7. There was blinding of all assessors who measured at least one key outcome	0	1	1	0	1	1	0	1
8. Measures of at least one key outcome were obtained from more than 85% of the subjects initially allocated to groups	1	1	1	1	1	1	1	1
9. All subjects for whom outcome measures were available received the treatment or control condition as allocated or, where this was not the case, data for at least one key outcome was analyzed by “intention to treat”	1	1	1	1	1	1	1	1
10. The results of between-group statistical comparisons are reported for at least one key outcome	1	1	1	1	1	1	0	0
11. The study provides both point measures and measures of variability for at least one key outcome	1	1	1	1	1	1	1	1
**Total**	7	10	10	8	11	8	4	7

In addition, for answering our leading questions in this meta-analysis, we performed four separate moderator variable analyses: (a) tremor assessment (clinical test vs. quantitative tremor assessment), (b) stimulation site (cerebellum vs. motor cortex), (c) stimulation session (single session vs. multiple sessions), and (d) sustained treatment effect (posttest: ≤ 1 day vs. retention test: > 1 day). Consistent with the conventional approaches to comparing the mean effect for different subgroups of comparisons [30], we used a Z-test using the mean and variance of the estimated effects. Further, we calculated *P*-value on differences in the estimated effects between two subgroups using the following three formulas. Given that the null hypothesis is that the true effect size is the same for both groups, *P* < .05 indicates that the treatment effect is significantly different between the two groups.

$$Diff=M_{B}-M_{A}\;\text{and}\;SE_{Diff}=sqrt\left(V_{A}-V_{B}\right)$$ (1)

$$Z_{Diff}=Diff/SE_{Diff}$$ (2)

$$P=2\left[1-\left((\Phi \left|Z_{Diff}\right|\right)\right]$$ (3)

where *M*_A_ is the estimated effect for group A, *M*_B_ is the estimated effect for group B, *V*_A_ is the variance of estimated effect for group A, *V*_B_ is the variance of estimated effect for group B, and Φ(*Z*) is the standard normal cumulative distribution.

### Measuring heterogeneity and publication bias

Heterogeneity between studies was tested using Cochran’s *Q*, *T*^2^, and Higgins and Green’s *I*^2^, and the prediction interval [33, 34]. These three procedures measured inconsistency in our meta-analysis and are important for interpreting the meta-analytic findings [33]. First, Cochran’s *Q* is a statistical test to determine whether the heterogeneity significantly exists based on a *P*-value. We used a *P*-value (< .10) for increasing the power for the significant test of heterogeneity between studies. Next, *T*^2^ is an estimate of variance of the true effects sizes in a random effects model. A value of *T*^2^ greater than 1.0 indicates substantial heterogeneity between studies. Third, *I*^2^ quantifies the percentage of heterogeneity between individual effect sizes used in the meta-analysis. This technique can show the proportion of the observed variance (i.e., heterogeneity) in the forest plot, and a value of *I*^2^ greater than 50% indicates substantial heterogeneity.

Publication bias was estimated using conventional meta-analysis approaches that include funnel plots and Egger’s regression test [30, 35, 36]. We followed three accepted steps: (1) create a funnel plot displaying the symmetry of the studies (i.e., standardized mean differences vs. standard error for each comparison), (2) apply the trim and fill technique and generate a subsequent funnel plot with imputed values for assessing an unbiased distribution, and (3) conduct Egger’s regression test for detecting the relation between actual effect sizes and standard error values (i.e., precision): a significant intercept (β_0_; *P* < .05) denotes high publication bias.

## Results

### Standardized mean difference effect

A random effects model meta-analysis on the 20 comparisons exhibited a significant overall standardized mean difference effect (effect size = 0.61; *SE* = 0.10; 95%CI = 0.42–0.79; *P* < 0.0001; Z = 6.38). This is a positive medium effect size according to Rosenthal and DiMatteo [37]. Fig 2 exhibits the 20 individual effect sizes that ranged from -0.27 to 1.52. The smallest (effect size = -0.27) and largest (effect size = 1.52) comparisons exceeded two *SD* beyond the overall standardized mean difference. Thus, after removing these two outliers, we conducted an additional analysis. The subsequent analysis showed a relatively similar overall effect size in comparison to the original findings (effect size = 0.58; *SE* = 0.09; 95%CI = 0.40–0.75; *P* < 0.0001; Z = 6.53). Overall, these findings indicate that non-invasive protocols reduced tremor symptoms in individuals with ET.

### Heterogeneity and publication bias

**Fig 3:**
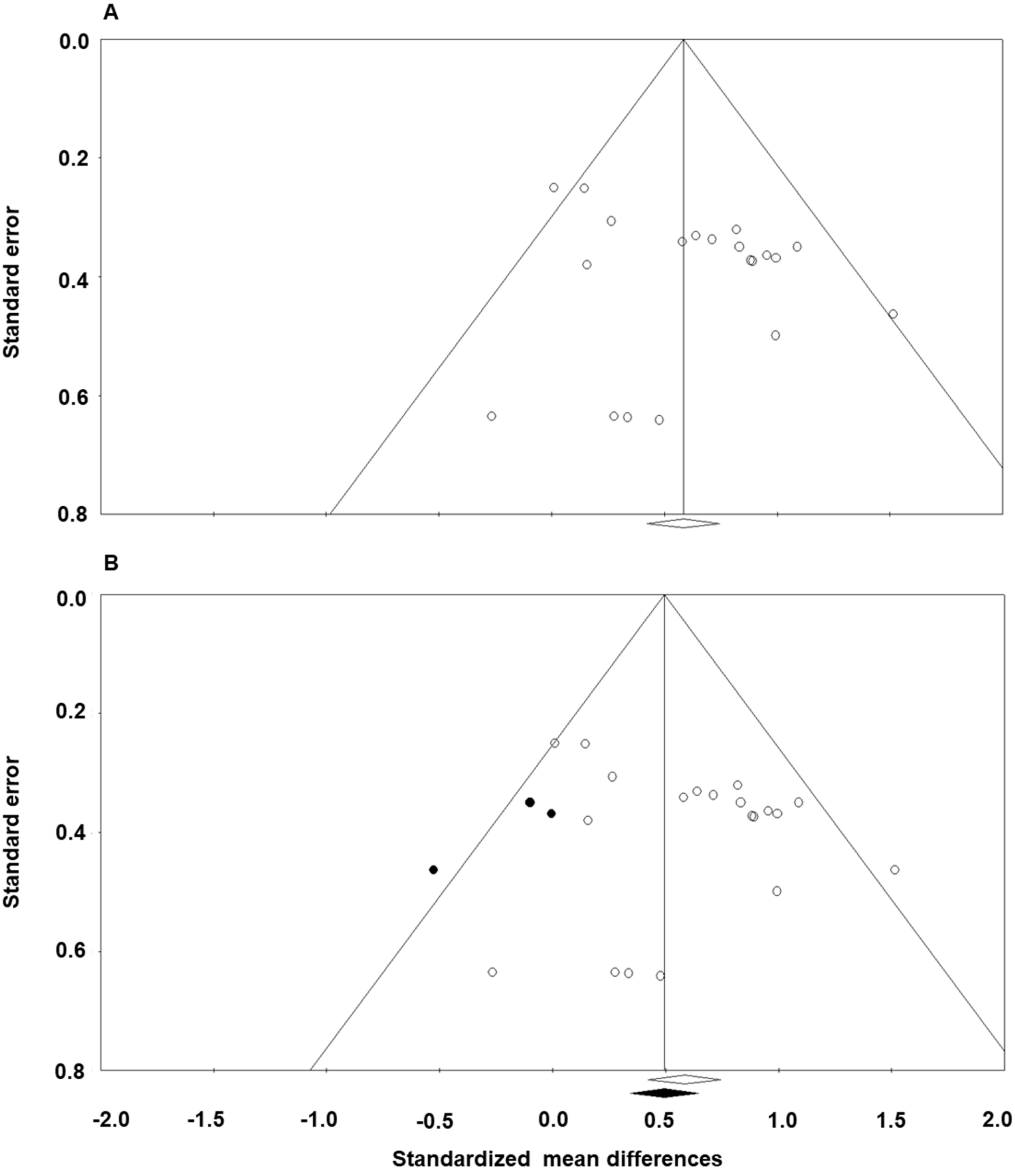
Funnel plots of the 20 comparisons for random effects model. (A) Original funnel plot and (B) Best estimate funnel plot of a symmetrical funnel unbiased effect. The x-axis represents the standardized mean difference and the y-axis indicates the standard error associated with each comparison. White circles and white diamond indicate our original 20 comparisons while the black circles and black diamond represent imputed comparisons after the trim and fill technique. A revised funnel plot after trim and fill method produced a symmetrical distribution with three imputed values. Relatively identical standardized effect sizes between the two plots (original effect size = 0.61 vs. revised effect size: 0.51) indicate minimal publication bias.

Heterogeneity measures between the 20 individual effect sizes revealed relatively low inconsistency between comparisons: (1) *Q* = 24.50 and *P* = 0.18, (2) *T*^2^ = 0.04, and (3) *I*^2^ = 22.43. For publication bias, Fig 3A displays an original funnel plot with moderately biased distribution of individual effects sizes against standard error values. The trim and fill method produced a revised funnel plot with a symmetrical distribution with three imputed values (i.e., black circles; Fig 3B) [36]. To estimate publication bias, we compared how the standardized effect size is shifted after adding the imputed values [30]. If the shift is trivial, we can assume that the publication bias is minimal. Our funnel plots displayed that the standardized effect sizes (i.e., original effect size: white diamond and revised effect size: black diamond) were relatively identical in both cases. In addition, Egger’s regression analysis showed an insignificant intercept (β_0_ = 1.09; *P* = 0.14) indicating no relation between the actual effect sizes and standard error (i.e., precision) [35]. Taken together, these findings confirmed that there was minimal publication bias in the 20 comparisons.

### Methodological quality

Table 4 displays methodological quality findings using the PEDro score for eight qualified studies. Six studies used sham stimulation against active stimulation during the experiment (Table 3), and five out of the six studies reported a random assignment for the active stimulation vs. sham stimulation groups (Table 4). The PEDro scores ranged from 4 to 11 (mean = 8.1 and *SD* = 2.2). Higher scores indicate better methodological quality for the study. These findings indicate relatively moderate quality across the eight studies in this meta-analysis.

### Moderator variable analyses

#### Tremor assessment

The first moderator variable analysis examined the effects of NIBS interventions on tremor symptoms as indicated by clinical tests and quantitative tremor assessment. This subgroup analysis identified two significant standardized effect sizes: (a) 11 clinical test comparisons: effect size = 0.54; *SE* = 0.12; 95%CI = 0.30–0.78; *P* < 0.0001; Z = 4.43; *T*^2^ = 0.01; *I*^2^ = 3.73% and (b) nine quantitative tremor assessment comparisons: effect size = 0.62; *SE* = 0.11; 95%CI = 0.40–0.84; *P* < 0.0001; Z = 5.56; *T*^2^ = 0.08; *I*^2^ = 42.20%. A Z-test showed that effect sizes between the clinical test and quantitative tremor assessment comparisons were comparable (*P* = 0.14). These findings indicate that NIBS interventions revealed positive clinical and subclinical effects on tremor in ET patients.

#### Stimulation site

In a second moderator variable analysis, we compared treatment effects of NIBS interventions between different targeted brain areas: (a) posterior cerebellum and (b) motor cortex: M1, preSMA, and premotor. The analysis revealed two significant standardized effect sizes: (a) 12 cerebellum comparisons: effect size = 0.55; *SE* = 0.11; 95%CI = 0.33–0.78; *P* < 0.0001; Z = 4.84; *T*^2^ = 0.04; *I*^2^ = 27.51% and (b) eight motor cortex comparisons: effect size = 0.75; *SE* = 0.17; 95%CI = 0.42–1.07; *P* < 0.0001; Z = 4.48; *T*^2^ = 0.02; *I*^2^ = 8.85%. A Z-test showed that effect sizes between the cerebellum and motor cortex comparisons were not significantly different (*P* = 0.12). These findings indicate that NIBS protocols inhibiting cortical excitation across the cerebellum and motor cortex reduced tremor symptoms in individuals with ET.

#### Session number

A third moderator variable analysis investigated how different numbers of stimulation sessions influenced a reduction of tremor. We categorized the number of sessions into (a) single session and (b) multiple sessions (i.e., 2–15 sessions). The subgroup analysis demonstrated two significant standardized effect sizes: (a) seven single session comparisons: effect size = 0.53; *SE* = 0.18; 95%CI = 0.18–0. 87; *P* = 0.003; Z = 3.01; *T*^2^ = 0.11; *I*^2^ = 52.22% and (b) 13 multiple sessions comparisons: effect size = 0.69; *SE* = 0.11; 95%CI = 0.47–0.91; *P* < 0.0001; Z = 6.12; *T*^2^ = 0.00; *I*^2^ = 0.00%. A Z-test showed that effect sizes between the single session and multiple sessions comparisons were comparable (*P* = 0.23). Both session number conditions similarly reduced tremor in ET patients.

#### Sustained treatment effect

The final moderator variable analysis determined whether the effects of NIBS interventions on tremor appeared at posttesting (≤ 1 day) or retention testing (> 1 day). This subgroup analysis revealed two significant standardized effect sizes: (a) 13 posttest comparisons: effect size = 0.62; *SE* = 0.12; 95%CI = 0.38–0.86; *P* < 0.0001; Z = 5.09; *T*^2^ = 0.07; *I*^2^ = 34.91% and (b) seven retention test comparisons: effect size = 0.61; *SE* = 0.16; 95%CI = 0.30–0.92; *P* < 0.0001; Z = 3.89; *T*^2^ = 0.001; *I*^2^ = 0.43%. A Z-test showed that effect sizes between posttest and retention test comparisons were not significantly different (*P* = 0.88). These findings indicate that NIBS interventions showed relatively short-term and long-term therapeutic effects on ET individuals.

## Discussion

The current systematic review and meta-analysis investigated the effects of NIBS interventions on tremor symptoms in ET patients. Each of the eight studies that used 1 Hz of rTMS, cTBS, or ctDCS protocols was included in this meta-analysis. Twenty total comparisons from the eight qualified studies were statistically synthesized, and the meta-analytic findings revealed that NIBS techniques reduced tremulous behaviors in individuals with ET. Moreover, the four moderator variable analyses demonstrated that the positive treatment effects of NIBS appeared across the following subgroups: (a) tremor assessment (clinical test vs. quantitative tremor assessment), (b) stimulation site (cerebellum vs. motor cortex), (c) session number (single session vs. multiple sessions), and (d) sustained treatment effect (posttest vs. retention test).

The overall significant standardized mean difference over 20 comparisons demonstrated the potential role of NIBS interventions as an ET treatment program. Moreover, two significant treatment effects on tremor estimated by clinical (effect size = 0.54) and subclinical (effect size = 0.62) tests support the hypothesis that NIBS approaches may contribute to minimizing tremor in ET patients [11]. These findings indicate that changes in tremor symptoms after NIBS protocols can be identified by both clinical aspects as well as quantitative measurements on upper limb movements (e.g., tremor amplitude of active and postural finger movements). However, a level of heterogeneity for the nine subclinical comparisons was relatively increased (*I*^2^ = 42.20%). Presumably, this variability between comparisons may be related to different experimental approaches such as device, task goal, or tremor magnitude calculations across studies.

Beneficial effects of NIBS interventions on ET patients were observed for two different stimulation sites that included the cerebellum (effect size = 0.55) and motor cortical (effect size = 0.75) regions, and these effects were comparable. All 20 comparisons used specific NIBS protocols for suppressing cortical excitation in either ipsilateral posterior cerebellum or contralateral motor cortex such as M1, premotor cortex, and pre-SMA. These NIBS protocols for ET patients assume two propositions: (a) increased tremor may be related to hyperactivity across the CTC network [38] and (b) decreasing activity in the critical regions along the CTC network such as the cerebellum and motor cortex may reduce tremulous behaviors [18]. Thus, the current subgroup meta-analytic findings indicate that cerebellar and motor cortical areas appear to be effective targets of NIBS interventions for minimizing ET symptoms.

However, neurophysiological mechanisms underlying the treatment effects of NIBS interventions on ET individuals are still unclear. Four out of the eight qualified studies in this meta-analysis reported brain activation patterns after NIBS treatments: (a) two cTBS on motor cortex and one cTBS on the cerebellum: motor cortical excitation using the amplitude of motor-evoked potentials (MEP) [24, 25, 27] and (b) one 1 Hz-rTMS on the cerebellum: resting state functional connectivity over the CTC network properties [29]. Common findings from three cTBS studies indicated that no lasting change in motor cortical excitation as indicated by MEP after stimulation for ET patients, whereas healthy controls showed suppressive effects of cTBS on motor cortical excitation. However, the 1 Hz-rTMS study reported increased functional connectivity across the cerebellar and motor cortical regions of the CTC network after stimulation treatment. These contrasting findings may be related to different types of stimulation, stimulation site, intensity, the number of sessions, and demographic or clinical characteristics of ET patients across individual studies [27]. Indeed, for ET patients short interval changes or even an absence of change in motor cortical excitation as indicated by MEP may be attributed to functional impairments in inhibitory cortical circuits frequently shown in neurodegenerative disease [39, 40]. Perhaps, the NIBS interventions in ET patients may facilitate an interaction between cerebellar and motor cortical regions leading to reduced responsiveness of pyramidal cells rather than acting on the corticospinal output cells [41].

For the number of intervention sessions, we found that both single session (effect size = 0.53) and multiple sessions (effect size = 0.69) of NIBS interventions exhibited positive therapeutic effects on ET. Importantly, individuals’ effect sizes for the multiple sessions demonstrated minimal heterogeneity across 13 comparisons (*I*^2^ = 0.00%). In this meta-analysis, four out of the eight qualified studies used multiple sessions of NIBS interventions that ranged from 2 to 15 sessions. Previous studies posited that repetitive stimulation protocols over time presumably induce better response in brain activity modulation, cognitive, and motor functions for individuals with neurological disease than a single session of stimulation [42–47]. However, given that improvements in tremulous behaviors were not linearly related to the number of sessions [42, 45], there may be a certain threshold in the number of sessions that optimizes the cumulative motor improvements. Taken together, repeated sessions of NIBS interventions may improve ET symptoms with minimal variability in comparison to a single session treatment [40].

A current subgroup analysis revealed comparable significant therapeutic effects across two different times of testing (posttest: ≤ 1 day and retention test: > 1 day) after stimulation. We included and analyzed seven comparisons from two studies [23, 29]. A mean interval between the baseline and retention test in this meta-analysis was 39.8 days (range: 7–84 days). Moreover, long-term lasting effects were observed in the studies that applied multiple sessions of NIBS interventions. Thus, long-term effects of NIBS interventions on ET symptoms may be related to the number of sessions; referred to as the likely dose-dependent effects [29, 48]. However, these meta-analytic findings should be carefully interpreted because of a limited number studies that reported changes in brain activation and behaviors at the long-term follow-up retention tests.

There are some limitations that need to be considered in this study. This meta-analysis included a limited number of studies that used each NIBS protocol for ET treatment. Further, specific stimulation protocols such as targeted brain areas and parameters (e.g., stimulation time and amplitude) are heterogeneous across studies. Finally, two studies did not include a sham stimulation protocol [28, 29].

Despite several limitations in this study, the significant treatment effects in this meta-analysis are the first quantitative evidence that suggests a potential role of NIBS as an alternative ET treatment option. Further, to advance the therapeutic effects of NIBS protocols on individuals with ET, we suggest the following important issues. First, the number of studies focusing on potential mechanisms underlying NIBS interventions as a treatment protocol is still lacking [11]. Additional studies are required to determine how NIBS interventions influence and reorganize properties of oscillating networks involved in reducing tremulous behaviors. Moreover, understanding possible confounders causing bias in NIBS treatment effects is crucial. For example, given that ET is a slow and age-related progressive disease [49], different severity symptoms of ET based on the age of disease onset and periods since disease diagnosis may affect brain modulation and motor improvement in response to NIBS stimulation [50]. Investigating tremor reduction after NIBS based on the different levels of symptom severity could be an effective way to confirm accurate treatment effects with minimal inter-individual variability.

In conclusion, this comprehensive systematic review and meta-analysis provided evidence that support positive effects of NIBS techniques on ET motor functions. Our subgroup analyses confirmed that these therapeutic effects were identified using both clinical and subclinical tests. Moreover, NIBS on cerebellar and motor cortical regions similarly revealed medium treatment effects. Future studies should examine brain reorganization patterns across the CTC tract after NIBS using structural and functional brain imaging, and determine how this brain modulation relates to a reduction of tremulous behaviors. In addition, if we conduct brain imaging scans for each individual with ET before stimulation, these efforts may contribute to developing personalized NIBS protocols and optimizing therapeutic effects of NIBS interventions. Finally, applying simultaneous stimulation on the bilateral side of the motor cortex and cerebellum or cortico-cerebellar regions within the ipsilateral hemisphere would increase the efficacy of NIBS treatment on ET patients [18, 51].

## Supporting information

S1 File PRISMA checklist. (DOC)
